# Transcorporeal decompression using a fully-endoscopic anterior cervical approach to treat cervical spondylotic myelopathy: surgical design and clinical application

**DOI:** 10.1186/s12891-022-06001-5

**Published:** 2022-11-30

**Authors:** Yanyan Ma, Zhijun Xin, Weijun Kong, Longsheng Zhang, Qian Du, Wenbo Liao

**Affiliations:** 1 Department of Spinal Surgery, The Affiliated Hospital of Zunyi Medical University, 149 Dalian Road, Huichuan District, Zunyi, 563099 Guizhou China; 2 Rehabilitation Department, Guizhou Provincial Orthopedics Hospital, Sixian street, Guiyang, 550007 China; 3 Orthopaedics, The Second Affiliated Hospital of Zunyi Medical University, Intersection between Xinpu Avenue and Xinlong Avenue, Zunyi, 563006 China

**Keywords:** Cervical spondylotic myelopathy (CSM), Decompression of spinal cord, Full-endoscopy, Minimally invasive surgery, Osteophyte

## Abstract

**Background:**

Anterior cervical discectomy and fusion (ACDF) is a common procedure for treating cervical spondylotic myelopathy (CSM), however, ACDF may cause pseudoarthrosis, accelerated degeneration of adjacent segments, loss of activity of fused segments and other complications. The full-endoscopic technique can treat CSM, without the aforementioned complications above. Therefore, it is of great clinical value to investigate the surgical scheme of anterior percutaneous full-endoscopic transcorporeal decompression of the spinal cord (APFETDSC).

**Methods:**

A total of 28 cases with single-segment Cervical spondylotic myelopathy (CSM) from April 2017 to July 2019 were involved in this study. The size of the disc-osteophyte complex was measured using imaging data prior to the operation. The diameter and direction of the bony passage was determined according to the size and central position of the complex, respectively. Twenty-eight patients underwent the above scheme for CSM. The clinical outcome evaluations included Visual Analog Scale (VAS) scores, Japanese Orthopedic Association (JOA) scores. The imaging assessment included MRI, CT and X-rays.

**Results:**

The diameter of the designed bony passage was about 6.9 mm, and directed toward the lower edge of the diseased lower vertebral body oblique to the center of the disc-osteophyte complex. All patients successfully completed the operation. The postoperative neck pain VAS and JOA were significantly improved compared to preoperative values (*p* < 0.01). Postoperative MRI indicated complete decompression of the spinal cord. CT scanning 1 year after the operation revealed an almost healed bony passage and X-ray imaging showed satisfactory physiological curvature of the cervical spine, without cervical instability.

**Conclusion:**

Based on the diameter and direction of the bony passage, as determined by the size and position of the disc-osteophyte complex, indicated by MRI and CT scanning, anterior percutaneous full-endoscopic transcorporeal decompression of the spinal cord offers good decompression of the spinal cord and ensures excellent therapeutic outcome.

**Supplementary Information:**

The online version contains supplementary material available at 10.1186/s12891-022-06001-5.

## Introduction

Cervical spondylotic myelopathy (CSM) refers to a series of clinical symptoms induced by the gradual loss of neurons and myelin sheath, caused by the degenerative alterations in cervical disc and secondary pathological alterations in adjacent structures, which include inflammation, hypertrophy, and proliferation [1, 2]. CSM patients are primarily characterized by gait instability, clumsy hands, motor weakness, and loss of feeling [3–5]. Patients with mild CSM are conservatively treated with physiotherapy, bed rest, and drug treatment. However, neurological deterioration should be monitored. Patients with severe clinical symptoms or progressive deterioration of neurological function should be treated in a timely surgical manner [2, 6, 7]. Anterior cervical discectomy and fusion (ACDF) directly depressurizes the spinal canal by removing the disc and osteophyte that protrude into the front of the spinal cord. This helps in achieving spinal cord functional recovery [6]. ACDF is a common surgical treatment for CSM, owing to its good clinical therapeutic outcome [6, 8]. Although ACDF can achieve interbody fusion, it may also lead to complications like pseudojoint, accelerated degeneration of adjacent segments, and loss of activity of fused segments [9–12]. Spine surgeons actively explore and improve their surgical methods to avoid these complications [13–15]. With the development of minimally invasive spinal surgeries, full-endoscopy is favored by spine surgeons due to its minimally invasive surgical procedure and satisfactory clinical outcome [16, 17]. Kong et al. [10] reported the successful completion of anterior percutaneous full-endoscopic transcorporeal decompression of the spinal cord (APFETDSC) on 32 patients with single-segment cervical CSM.

The fundamental technical problem associated with APFETDSC is the establishment of a bony passage with appropriate size and direction. If the bony passage is too large, it may result in the collapse of the vertebral body. Conversely, if the bony passage is too small, the disc-osteophyte complex may not be completely removed [18]. In this study, the diameter and direction of the bony passage were designed, using imaging, by measuring the size and central position of the disc-osteophyte complex, respectively. The disc-osteophyte complex was exposed entirely and resected under a full-endoscopy, and the damage to the vertebral body and disc were minimized. The aim of this study was to design the surgical scheme of anterior percutaneous fully-endoscopic transcorporeal decompression of the spinal cord (APFETDSC) using cervical disc-osteophyte complex resection, and to evaluate its clinical therapeutic outcome.

## Methods

### Patient selection

The study was approved by the ethics committee. Informed consent was obtained from all patients. The inclusive criteria were as follows: 1) CSM patients with a single-segment disc-osteophyte complex; 2) no improvements after 3 months of standardized conservative treatment; 3) A combination of imaging, symptoms, and signs diagnosis of CSM patients, confirmed by a combination of assessments; 4) no cervical instability; 5) no less than 12 months of follow-up. The exclusive criteria were as follows: 1) cervical instability; 2) ligamentum flavum or simple posterior compression; 3) compression of two or more spinal cord segments; 4) coagulation dysfunction; 5) intervertebral infection; 6) cervical deformity and anterior cervical surgical history.

According to the symptoms，during April 2017 and july 2019， there are 28 patients that meet the standards to be listed. Totalling 17 males and 11 females who are required to finish their follow-ups. Tables 1 and 2 summarizes the characteristics of these patients. The study was performed after obtaining due permission from the local institutional review board as well as informed consent from the patients.

**Table 1:** Clinical features and affected level (*n* = 28)

Basic Features	Number of patients(%)
Preoperative clinical features and signs	
Neck pain	25 (89.29%)
Numbness in hands and decreased mobility	20 (71.42%)
Weakness of lower limbs	18 (64.29%)
Hoffman positive	8 (28.57%)
Dysuria	20 (71.43%)
Knee hyperreflexia	16 (57.14%)
Diseased segment	
C3/4	2 (7.14%)
C4/5	3 (10.71%)
C5/6	15 (53.57%)
C6/7	8 (28.57%)

**Table 2:** Clinical characteristics of patients

Parameters	Number
Gender	
Male	17
Female	11
Age (yeas), mean ± s.d.	59.25 ± 11.36
Minimum-Maximum	42-76
BMI (kg/m^2^), mean ± s.d.	24.23 ± 2.53
Minimum-Maximum	21.73-26.45
Follow-uptime (month), mean ± s.d.	13.85 ± 1.86
Minimum-Maximum	12.84-15.52
Duration of symptoms (month), mean ± s.d.	14.29 ± 4.64
Minimum-Maximum	9.56-17.45

### Size of the disc-osteophyte complex

MRI and CT data of the cervical spine from 28 CSM patients with single-segment disc-osteophyte complex were obtained. The size of the disc-osteophyte complex was measured using IMPAX Client 16 (Siemens, Germany).

#### Measurement of width

**Fig. 1:**
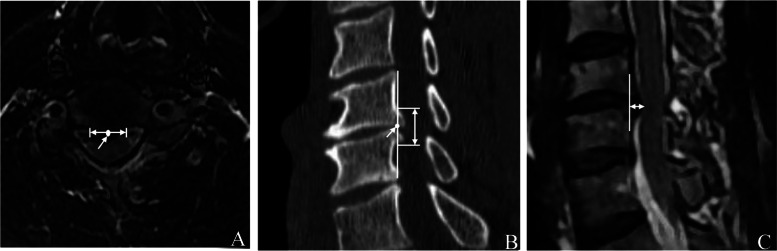
The measurement method of the width of the disc-osteophyte complex on MRI,The center point of the width was the center of the transverse position (**A**); The measurement method of the height of the disc-osteophyte complex on CT, The center point of the height was regarded as the center of the sagittal lesion position (**B**); The measurement method of the depth of the disc-osteophyte complex on MRI (**C**); (white double arrow)

Using the most severe level of spinal cord compression in the MRI cross-section of the lesion space, the linear distance between the disc-osteophyte complex and the left and right intersection of the posterior longitudinal ligament was determined as the width. The center point of the width was the center of the transverse position (see Fig. 1a).

#### Measurement of height

Using the CT sagittal plane with the most prominent disc-osteophyte complex, the linear distance between the line connecting the posterior upper edge of the upper vertebral body and the posterior lower edge of the lower vertebral body in the lesion space to the upper and lower intersection of the disc-osteophyte complex, respectively, was determined as the height. The center point of the height was regarded as the center of the sagittal lesion position (see Fig. 1b).

#### Measurement of depth

Using the sagittal MRI plane with the most severe spinal cord compression, the line connecting the midpoint of the posterior edge of the upper vertebral body to the midpoint of the posterior edge of the lower vertebral body was set as the standard line. The vertical distance between the vertex of the disc-osteophyte complex and the standard line was determined as the depth (see Fig. 1c).

### Cervical spine stability assessment

Assessment of cervical spine stability using the Cobb angle. The Cobb angle four-line measurement was carried out in which the first line was parallel to the end plate of C2, the second line was parallel to the end plate of C7, two vertical lines were drawn for the above two lines, and the acute angle between the two perpendicular lines was the Cobb angle [17] (see Fig. 7d).

### Surgical design

**Fig. 2:**
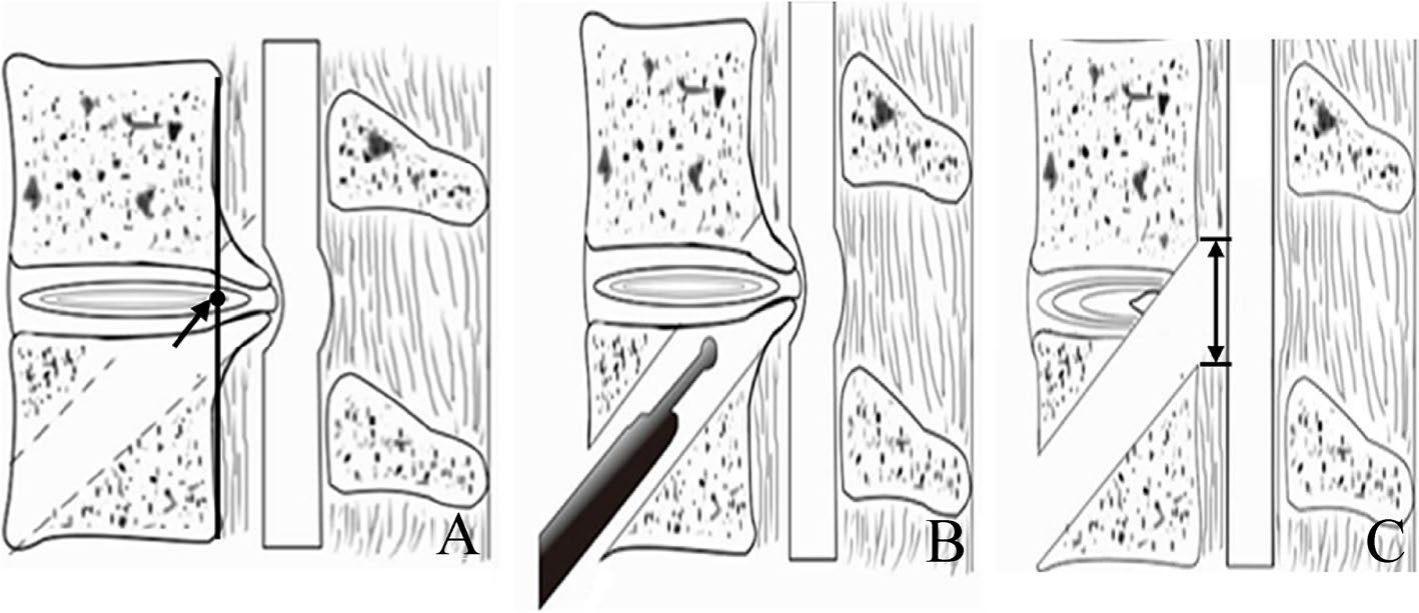
Artistic illustration of bone channel establishment. The bone channel was established at the anterior lower edge of the vertebral body under the diseased segment obliquely toward the center of the disc-osteophyte complex (**A**); the endoscopic system is inserted through the bone channel (**B**); The disc-osteophyte complex was fully exposed and completely removed to complete the decompression of the cervical spinal canal, the left and right distances of the maximum decompression range were set as the decompression width (black double arrow) (**C**)

We designed the bony passage with a diameter of 6.9 mm, based on the height, width, and depth of the disc-osteophyte complex. We next exposed and removed the disc-osteophyte complex, using a fully-endoscopic approach. The center point of the width of the disc-osteophyte complex (see Fig. 1a) and the center point of the height (see Fig. 1b) were set as the center position of the lesion. Next, we made an incision from the front and lower edge of the lower vertebral body of the diseased segment oblique to the center position, from anterior-inferior to posterior-superior (see Fig. 2).

### The fully-endoscopic instrument

The spinal endoscopic system (SPINENDOS GmbH, Germany) included an endoscope with an angle range of 30°, a 4.3 mm working channel, an irrigation system, a 6.9 mm outer sheath, associated surgical instruments, a Gimmi-SPINENDOS digital camera system, and a low-temperature radio frequency system (ArthroCare Corporation，California, USA).

### Surgery

**Fig. 3:**
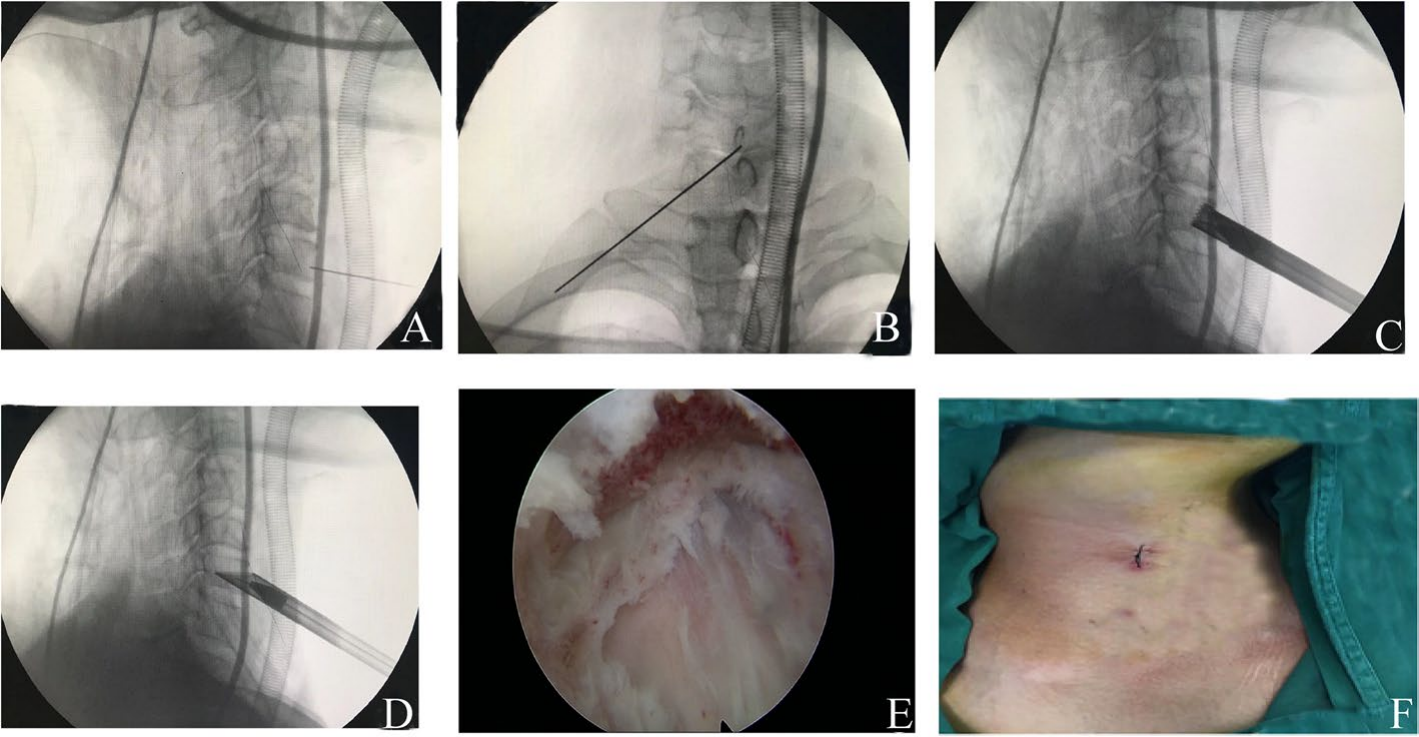
The C-arm fluoroscopy showed that the Kirschner wire was located in the middle of the lower edge of the lesion, and the lateral direction was oblique to the posterior upper edge of the lower vertebral body at the lesion site (**A**, **B**); The bone channel is established with the assistance of the C-arm using the ring perimeter (**C**); The working trocar is installed and the endoscopic operating system is placed (**D**); The dural sac is visible after decompression (**E**); The incision is sutured (**F**)

All patients received gastric tube insertion prior to the operation and general anesthesia using endotracheal intubation. Patients were placed in a supine position with the neck slightly extended. 15 mg of hydrogen iodide was injected into the gastric tube to show the position of the esophagus under C-arm fluoroscopy. Preoperative CT and MRI images determined the position of the disc-osteophyte complex and the trajectory of bony passage. During the operation, the horizontal vertebral segment of the diseased spine was identified and marked using C-arm positioning. Routine disinfection was performed and a sterile sheet was paved. Using the two-finger puncture method, the space between the carotid and visceral sheaths was separated along with the medial sternocleidomastoid muscle and Kirschner wire was used for percutaneous puncture to locate the lower edge of the lower vertebral body at the lesion site. The C-arm fluoroscopy showed that the Kirschner wire was located in the middle of the lower edge of the lesion, and the lateral direction was oblique to the posterior upper edge of the lower vertebral body at the lesion site (see Fig. 3 a and b). The skin was cut about 6 mm, and the primary, secondary, and tertiary dilators were placed along the Kirschner wires. Based on the working channel, essential tissues were bluntly separated in the working area. Next, different levels of dilators were successively removed and the dilating rods and ring were inserted along the Kirschner wires. The bony passage was established from the anterior lower to the posterior upper vertebral body to the central point, based on the ring (see Fig. 2a). Under C-arm fluoroscopy, once the ring reached the posterior wall of the lower vertebral body at the lesion site, the ring was removed and the bone strip was cut (see Fig. 3c). The working sleeve was installed and the endoscopic operating system was inserted into the working sleeve. Subsequently, the endoscopic decompression procedure was initiated (see Fig. 3d). Intraoperative continuous saline irrigation was conducted to facilitate a clear surgical vision. Under endoscopy, the protruding osteophyte was polished using a bone minimally invasive dynamic system, and the nucleus pulposus with the protruding disc was removed. A partial posterior longitudinal ligament was removed and the dural sac was identified after decompression (see Fig. 3e). The spinal cord and nerve were decompressed completely. Upon confirmation of no active bleeding in the spinal canal and passage, the allogeneic bone strip was placed in the bony passage, followed by the removal of the endoscopic operating system. Since there was no active bleeding, no drainage tube was required, and the incision was sutured and covered with sterile dressing (see Fig. 3f). On the first day post operation, patients were provided with a protective neck brace and were allowed to be mobile. Patients were discharged 2-4 days after the operation.

**Fig. 4:**
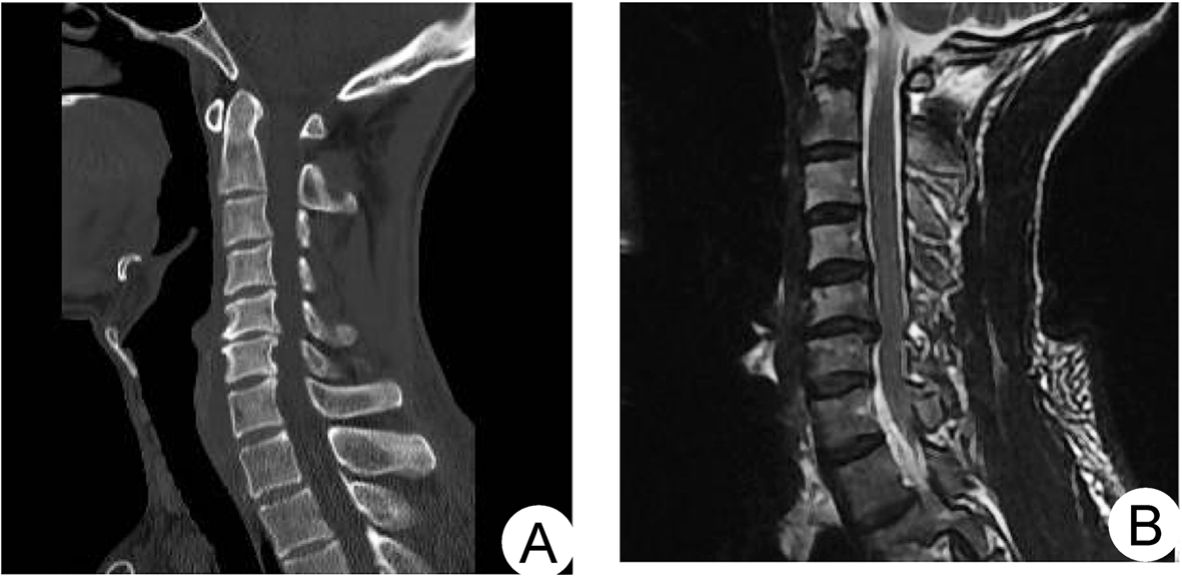
Preoperative CT images showed the formation of a disc-osteophyte complex in the cervical spine (**A**); preoperative MRI sagittal images showed significant compression of the spinal cord by the disc-osteophyte complex (**B**)

A typical case: a 42-year-old male patient presented with neck and right upper limb pain and numbness. He had C5-6 disc-osteophyte complex protruding into the spinal canal, resulting in spinal canal stenosis and spinal cord compression (see Fig. 4).

### Clinical evaluation

The degree of patient neck pain was evaluated using the pain visual analogue scale (VAS) and the neurological function was assessed by the Japanese Orthopaedic Association Score (JOA). VAS and JOA scores at 1 week, 3 months, 6 months, and 12 months after the operation were recorded and compared with preoperative scores. Excellent and good rates were evaluated using a modified Macnab score at the last follow-up.

**Fig. 5:**
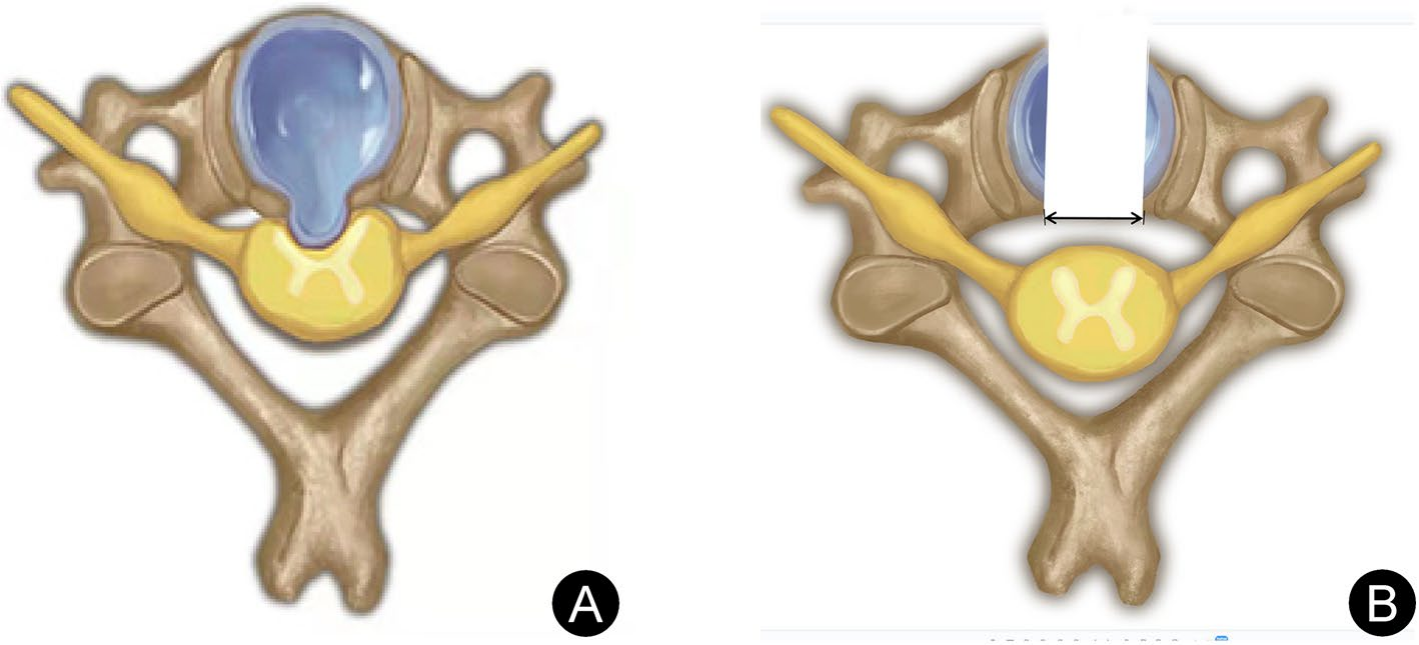
Schematic diagram for measuring the extent of decompression of the intervertebral disc complex. Compression of the spinal cord by f a disc-osteophyte complex; the left and right distances of the maximum decompression range were set as the decompression width (**B**)

The width and height of decompression were measured using the IMPAX Client 16 software (Siemens, Germany), based on the postoperative CT data of the cervical spine of CSM patients. Using the CT cross-section with the most extensive decompression range, the left and right distances of the maximum decompression range were set as the decompression width (see Fig. 5). The upper and lower distance of the maximum decompression range were set as the decompression height (see Fig. 2C).

The spinal cord decompression was identified 1 week after the operation using cervical MRI. Bony passage healing was identified 1 year after the operation using cervical CT scanning. Cervical flexion and extension X-rays were performed 6 months after the operation to evaluate stability and mobility of the cervical spine.

### Statistical analysis

All statistical analyses were performed with the SPSS18.0 software. The measurement data are expressed as mean ± standard deviation. Paired t-test was used for pairwise comparison. *P* < 0.05 was considered statistically significant.

## Results and analysis

### Imaging and bony passage design results

**Fig. 6:**
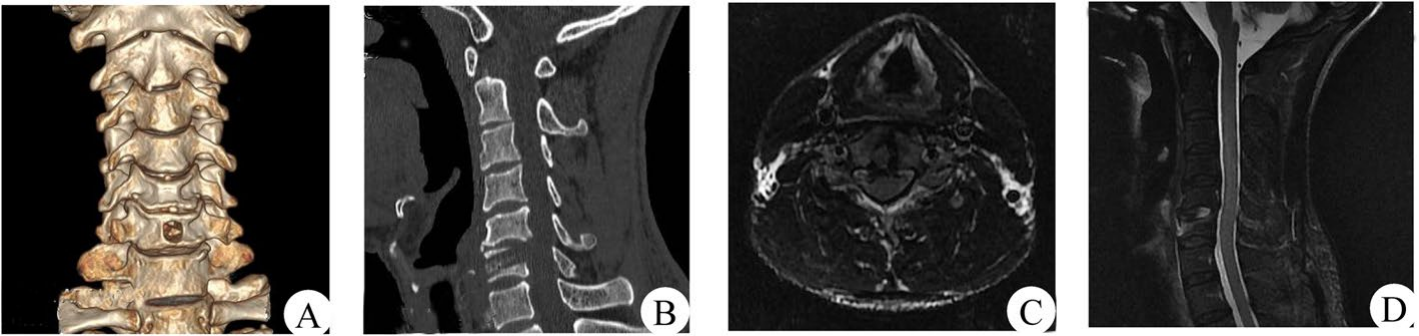
Ostoperative CT showed that the disc-bone complex was removed and 3D reconstruction showed intact vertebral tunnels with no loss of vertebral height and no fracture occurred (**A**, **B**). MRI showed adequate spinal cord decompression (**C**, **D**)

**Fig. 7:**
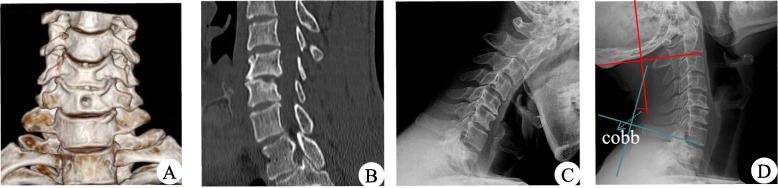
One year after the operation, CT scanning showed that the bony passage healed well and X-ray indicated that the physiological curvature of the cervical spine remained intact, with no cervical instability

The disc-osteophyte complex had a height of 8.17 ± 1.34 mm (range = 6.5-10.5 mm), width of 9.27 ± 0.83 mm (range = 7.8-10.6 mm), and depth of 4.61 ± 0.82 mm (4.4-6.8 mm). The diameter of the designed bony passage was about 6.9 mm, directed toward the lower edge of the lower vertebral body of the diseased spinal segment oblique to the center position of the disc-osteophyte complex. The decompression area had a height of 9.80 ± 0.62 mm (range = 8.2-10.5 mm), and width of 10.4 ± 0.96 mm (range = 9.0-11.7 mm). Following the operation, there was no evidence of residual disc-osteophyte complex and postoperative MRI revealed complete decompression of the spinal cord. One year after the operation, CT scanning showed that the bony passage healed well and X-ray indicated that the physiological curvature of the cervical spine remained intact, with no cervical instability (see Figs. 6 and 7).

Table 2 summarises the Cobb angle during the follow-up period. The Cobb angle at 1 week postoperatively was not significantly different from the preoperative one, indicating no postoperative cervical instability. The difference in Cobb angle between 1 week postoperatively and 3 months postoperatively and between 12 months postoperatively and 6 months postoperatively were significantly different, suggesting that some patients may have improved cervical spine curvature.

### Clinical results

All patients successfully completed the operation. The average operation time was 118.35 ± 12.95 min, the average hospital stay was 3.53 ± 0.57 days, and the average follow-up time was 13.85 ± 1.86 months. The average VAS and JOA scores during follow-ups are summarized in Table 3. The excellent and good rates in the last follow-up were 89.28%.The average blood loss was 27.89 ± 3.55 ml.preoperative haemoglobin volume (g/L): 125.07 ± 20.04, postoperative haemoglobin volume (g/L): 123.05 ± 18.4, the difference was not statistically significant.

**Table 3:** Comparison of VAS scores, JOA and Cobb score between pre and post-operation (*n* = 28, $\overline{\textrm{x}}\pm \textrm{s}$)

	M ± SD		M difference	95% Confidence Interval		P
				Lower	Upper	
VAS (preo-posto_1week)	5.71 ± 0.60	3.29 ± 0.46	2.42	2.14	2.71	0.000
VAS (posto_1week-posto_3months)	3.29 ± 0.46	2.11 ± 0.68	1.17	0.84	1.51	0.000
VAS (posto_3months-posto_6months)	2.11 ± 0.68	1.36 ± 0.55	0.75	0.36	1.14	0.001
VAS (posto_6months-posto_12months)	1.36 ± 0.55	0.82 ± 0.39	0.53	0.26	0.80	0.000
JOA (preo-posto-1 week)	6.89 ± 1.13	11.61 ± 1.16	−4.71	−5.28	−4.14	0.000
JOA (posto_1week-posto_3months)	11.61 ± 1.16	12.71 ± 1.08	−1.10	−1.76	−0.44	0.002
JOA (posto_3months-posto_6months)	12.71 ± 1.08	13.07 ± 0.53	−0.35	−0.79	0.08	0.106
JOA (posto_6months-posto_12months)	13.07 ± 0.53	13.82 ± 0.39	−0.75	− 0.97	− 0.52	0.000
C2–7 Cobb score (preo-posto_1week)	8.60 ± 0.70	8.14 ± 0.50	0.18	−0.07	0.44	0.152
C2–7 Cobb score (posto_1week-posto_3months)	8.14 ± 0.50	9.10 ± 0.49	−0.97	−1.19	− 0.74	0.000
C2–7 Cobb score (posto_3months-posto_6months)	9.10 ± 0.49	9.23 ± 0.52	−0.12	−0.27	0.02	0.091
C2–7 Cobb score (posto_6months-posto_12months)	9.23 ± 0.52	10.11 ± 0.72	−0.87	−1.24	−0.50	0.000

## Discussion

This study found that the use of APFETDSC for the treatment of spinal cervical spondylosis by preoperatively determining the orientation of the bone channel had better clinical results and provided a new surgical approach for the treatment of spinal cervical spondylosis. CSM pathogenesis originates from degenerative disc diseases that lead to increased facet stress, followed by osteophyte formation [4]. The mechanism of osteophyte formation remains elusive. Continuous abnormal movement causes local inflammation which may lead to osteophyte formation [19]. CSM is usually caused by the backward protrusion of osteophyte and disc, which reduces the sagittal diameter of the spinal canal. Thus, the spinal cord or its blood vessels become directly compressed, resulting in compression or ischemia of corresponding segments of the spinal cord, followed by spinal cord dysfunction, and corresponding clinical signs and symptoms [20]. CSM symptoms are mostly related to the disc-osteophyte complex compressing the spinal cord [5, 21]. Surgical treatment aims to completely remove the compression of the disc-osteophyte complex on the spinal cord and restore normal physiological curvature, which ultimately helps in restoring spinal cord function and preventing disease development or deterioration [22, 23].

Completion of protruding disc-osteophyte complex resection is an essential reference index for evaluating the curative effect of the operation. In sagittal MRI images, some scholars suggested that the connecting line from the midpoint of the posterior edge of the upper vertebral body to the midpoint of the posterior edge of the lower vertebral body at the diseased segment represents the standard line, and the distance between the standard line and the disc-osteophyte complex is the size of the disc-osteophyte complex [21, 24]. Nevertheless, the disc-osteophyte complex is a three-dimensional structure and a single dimension cannot accurately evaluate its size. Based on the measured height, width, and depth of the disc-osteophyte complex, we should estimate its size relatively accurately. MRI can directly reveal the location and severity of spinal cord lesions, but it is not sensitive to bone cortex [3, 25]. Yu et al. [26] indicated that CT images can fully identify osteophyte and calcified disc. Using the most apparent CT sagittal plane of disc-osteophyte complex, the distance between the connecting line from the posterior upper edge of the upper vertebral body to the posterior lower edge of the lower vertebral body at the diseased segment and the upper and lower intersection of disc osteophyte complex can be determined as its height. MRI clearly reveals the protruding morphology of the disc and its relationship with the dural sac, nerve root and other surrounding tissues [27], thereby making this the most accurate method for clinically evaluating the integrity of the disc [28]. In terms of CSM patients, the spinal cord is usually most compressed at the protruding disc. In this paper, we measured the width and depth of the disc-osteophyte complex using MRI images. The size of the disc-osteophyte complex removed during operation was similar to that measured before operation. Therefore, the height, width, and depth of the disc-osteophyte complex measured by imaging accurately reflected its size and compression degree, providing a vital reference for the surgery.

Although the disc-osteophyte complex sizes in this study were different, most were within 10.5 mm in width and 10.6 mm in height, based on imaging. The diameter of the bony passage designed prior to the operation was about 6.9 mm, and it was directed toward the anterior lower edge of the lower vertebral body of the diseased segment oblique to the center position of the disc-osteophyte complex. Twenty-eight patients successfully completed the operation in this study. This involved the complete removal of the disc-osteophyte complex. Although the size of the bony passage was only 6.9 mm, its decompression range height and width reached 10.5 and 11.7 mm, respectively. Thus, the disc-osteophyte complex in patients with common CSM can be removed. This is because the direction of the designed bony passage is inclined, and the decompression range is expanded and the damage to disc is reduced by rotating and adjusting the position during operation. At the last follow-up, the clinical effect was valid, without any surgery-related complications. The clinical outcome was similar to that reported in the treatment of CSM patients using anterior disc resection and fusion [6].

With the continuous improvement of endoscopic and surgical instruments, the indications of a fully-endoscopic surgery are ever expanding [17, 29]. Chen et al. [18] performed fully-endoscopic anterior transcorporeal cervical discectomy to treat cervical disc herniation. During the postoperative follow-up, the bony passage healed completely and the spinal cord was fully decompressed. Du et al. [30] also performed fully-endoscopic transcorporeal procedure to treat cervical disc discectomy. All patient symptoms were significantly improved and the bony passage healed well, without any surgery-related complications. Similarly, there were no signs of hematoma, nerve root injury, or incision infection in the 28 CSM patients treated with APFETDS in this study. Postoperative reexamination of the cervical MRI revealed that the spinal cord was basically decompressed. CT scans from 1 year after the operation showed that the bony passage healed well and X-ray images showed regular cervical spine activity. The bony passage was obliquely directed from the lower edge of the lower vertebrae of the diseased segment to the center of the disc-osteophyte complex. In terms of the sufficient removal of proliferative osteophyte and sufficient decompression of the spinal cord, damages to local tissues were markedly reduced, decreasing the possibility of postoperative cervical instability.

There were certain limitations to our study. First, there are Insufficient number of cases included in the study, and the follow-up time was not very long. In addition, the long-term efficacy of the surgery requires further verification, with longer follow-up. Second, this study was a single-center study, and the experience of the surgeon and his personal preferences may cause differences in the results. Third, this study only conducted this technology to treat single segment CSM patients. Therefore, our relevant surgical indications were limited. With the comprehensive future development of full-endoscopies, we hope that the application of this technology can be further extended to 2-3 segment CSM patients.

## Conclusions

Based on the diameter and direction of the bony passage, as determined by the size and position of the disc-osteophyte complex, indicated by MRI and CT scanning, anterior percutaneous full-endoscopic transcorporeal decompression of the spinal cord offers good decompression of the spinal cord, and ensures excellent therapeutic outcome. Hence, it holds great clinical value, and deserves much attention in the future.

## Supplementary Information

**Additional file 1.**

## Data Availability

All data generated or analysed during this study are included in this published article.

## Abbreviations

ACDF: Anterior cervical discectomy and fusion
CSM: Cervical spondylotic myelopathy
APFETDSC: Anterior percutaneous full-endoscopic transcorporeal decompression of the spinal cord
