# Correction: Transcorporeal decompression using a fully-endoscopic anterior cervical approach to treat cervical spondylotic myelopathy: surgical design and clinical application

**DOI:** 10.1186/s12891-023-06180-9

**Published:** 2023-01-25

**Authors:** Ma Yanyan, Xin Zhijun, Kong Weijun, Zhang Longsheng, Du Qian, Liao Wenbo

**Affiliations:** 1 Department of Spinal Surgery, The Affiliated Hospital of Zunyi Medical University, 149 Dalian Road, Huichuan District, Zunyi, 563099 Guizhou China; 2 Rehabilitation Department, Guizhou Provincial Orthopedics Hospital, Sixian street, Guiyang, 550007 China; 3 Orthopaedics, The Second Affiliated Hospital of Zunyi Medical University, Intersection between Xinpu Avenue and Xinlong Avenue, Zunyi, 563006 China

**Correction: BMC Musculoskelet Disord 23, 1031 (2022)**

10.1186/s12891-022-06001-5

Following publication of the original article [1], the authors corrected the sentence “Table 2 summarises the Cobb angle during the follow-up period” to “Table 3 summarises the Cobb angle during the follow-up period” in the Results and analysis section.

The original article [1] has been updated.

## References

[1] Ma Y, Xin Z, Kong W (2022). Transcorporeal decompression using a fully-endoscopic anterior cervical approach to treat cervical spondylotic myelopathy: surgical design and clinical application. BMC Musculoskelet Disord.

